# Human Bocavirus in Tonsillar Lymphocytes

**DOI:** 10.3201/eid1408.080300

**Published:** 2008-08

**Authors:** Xiaoyan Lu, Linda R. Gooding, Dean D. Erdman

**Affiliations:** * Centers for Disease Control and Prevention, Atlanta, Georgia, USA; † Emory University School of Medicine, Atlanta

**Keywords:** bocavirus, lymphocytes, tonsil, adenoid, respiratory viruses, letter

**To the Editor:** We read with great interest the recent report by Longtin and colleagues (*1*) describing human bocavirus (HBoV) infection among Canadian children with acute respiratory tract illnesses (ARI). The authors identified HBoV by PCR in nasopharyngeal aspirates from 13.8% of young children hospitalized with ARI, an infection rate well within the range reported by other studies on children (*2*). However, these authors also detected an unexpectedly high rate of HBoV for their control group (43%), >3 times the rate for ARI case-patients. In contrast, several similar studies did not detect HBoV in asymptomatic children. Kesebir et al. detected HBoV in 23 (5.2%) of 425 young children with ARI but in none of 96 children during routine well-child visits (*3*). Maggi et al. detected HBoV in 9 (4.5%) of 200 infants with ARI but in none of 30 healthy infants or 21 preadolescent healthy children without signs of ARI or history of asthma or wheezing (*4*). Finally, Allander et al. detected HBoV in 5 (19%) of 259 young children with acute expiratory wheezing but in none of 64 children who had not had respiratory symptoms during the preceding 4 weeks (*5*). In a recent study, we detected HBoV by PCR in 44 (12%) of 369 Thai children <4 years of age hospitalized with pneumonia but in only 2 (2%) of 85 asymptomatic age and temporally matched controls (*6*).

The inexplicably high rate of HBoV infection for patient controls reported by Longtin et al. (*1*) may reflect a unique feature of the children selected. The 100 controls were children without concomitant respiratory symptoms or fever at admission who were hospitalized during the study period for elective surgery, primarily of the ear, nose, and throat (71%). Most surgeries consisted of myringotomies, adenoidectomies, and tonsillectomies. The authors reported that these surgeries were more frequently performed on children found to be PCR positive for HBoV than on children negative for HBoV (84% vs. 61%). One possible explanation is that HBoV infection may directly induce inflammation of tonsillar tissues or facilitate bacterial superinfection prompting surgical intervention. Another possibility is that inflammation of mucosal lymphoid tissues enhances HBoV replication by recruitment of immune cells permissive for HBoV infection or by latent virus reactivation. Persistent infections and dependence on rapidly dividing cells are common features of the related parvoviruses, for example, human erythrovirus B19 (*7*). The presence of low-level persistent HBoV infections may also help explain the exceptionally high rates of respiratory viral co-infections found with HBoV, as high as 90% in 1 study (average 42%) (*2*,*6*). Longtin et al. (*1*) found that 71% of the HBoV-positive patients were also co-infected with another respiratory virus. As we previously hypothesized (*6*), co-virus–induced cellular damage resulting in high levels of cellular division and differentiation may stimulate HBoV reactivation and replication, as has been shown for polyomavirus following induced kidney damage in newborn mice (*8*).

To assess whether HBoV is present in tonsillar lymphocytes and therefore possibly explain the high rate of PCR positives obtained by Longtin et al. (*1*) in their patient controls, we tested DNA extracts of lymphocytes separated by Ficoll-Paque from nasopharyngeal tonsils or adenoids (AL) and palatine/lingual tonsils (TL) from 164 patients (mostly children) undergoing routine adenoidectomies and tonsillectomies for HBoV DNA. Of these children, 21 had AL only, 57 had TL only, 18 had both AL and TL collected separately, and 68 had AL and TL combined in 1 container. Hypertropic tonsil and adenoid tissues were removed because of clinical complications, generally obstructive sleep apnea, otitis media, or chronic tonsillitis. Data were not available on whether the children had concurrent respiratory tract illness, although surgeries would likely be postponed if symptoms were apparent. The median age of 162 children for whom age data were available was 5 years (range 1–19.7 years). HBoV real-time PCRs were performed as previously described, targeting 2 unique regions of the HBoV genome (*9*). All extractions were performed in a separate laboratory from PCR activities, and negative-assay controls were included in all PCR runs to monitor for DNA contamination.

HBoV DNA was detected by both PCRs in lymphocytes from 53 (32.3%) children (median age 3.7 years, range 1–7.6 years). A single assay target (nonstructural protein gene NS1 or NP–1) was positive in specimens from 6 additional children (3.7%), but these specimens were classified as HBoV negative because they did not meet our strict criteria for a positive test result. Children PCR negative for HBoV were significantly older (median age 5.5 years, range 1.8–19.7 years; p<0.001). HBoV was more often detected in AL (56%) than TL specimens (16%; p<0.001); the age distributions for children from each group were similar. Among 12 HBoV-positive children from whom both AL and TL were available and collected in separate containers, 6 were positive for HBoV in both specimens, 4 were positive for AL alone, and 2 were positive for AL but only for a single PCR assay target. Moreover, HBoV was present at a substantially higher load in AL (median 3.1 × 10^5^ copies/10^7^ cells; range 2.8 × 10^1^ to 1.2 × 10^9^ copies/10^7^ cells) than TL (median 1.6 × 10^3^ copies/10^7^ cells; range 0.1 × 10^0^ to 5.3 x10^7^ copies/10^7^ cells) as measured by quantitative PCR (*9*). We have no clear explanation for the relative predominance of HBoV DNA in AL. The adenoids are located at the back of the nasopharnyx in close proximity to inhaled pathogens and are covered with ciliated pseudostratified epithelium (respiratory epithelium) that may better support primary virus replication than the palantine and lingual tonsils, which are located in the lower pharynx and covered with nonkeratinized, stratified, squamous epithelium.

Our findings suggest that HBoV may establish latent or persistent infections of mucosal lymphocytes or contribute to tonsillar hyperplasia in young children. Further studies with appropriately matched controls will be necessary to fully understand the nature of HBoV infection and its role in human disease**.**
